# Affective Computing on Machine Learning-Based Emotion Recognition Using a Self-Made EEG Device

**DOI:** 10.3390/s21155135

**Published:** 2021-07-29

**Authors:** Ngoc-Dau Mai, Boon-Giin Lee, Wan-Young Chung

**Affiliations:** 1 Department of Artificial Intelligence Convergence, Pukyong National University, Busan 48513, Korea; ngocdaumai95@pukyong.ac.kr; 2 School of Computer Science, The University of Nottingham Ningbo China, Ningbo 315100, China; boon-giin.lee@nottingham.edu.cn

**Keywords:** electroencephalogram (EEG), affective computing, emotion recognition, entropy measures, support vector machine (SVM), multi-layer perceptron (MLP), one-dimensional convolutional neural network (1D-CNN)

## Abstract

In this research, we develop an affective computing method based on machine learning for emotion recognition using a wireless protocol and a wearable electroencephalography (EEG) custom-designed device. The system collects EEG signals using an eight-electrode placement on the scalp; two of these electrodes were placed in the frontal lobe, and the other six electrodes were placed in the temporal lobe. We performed experiments on eight subjects while they watched emotive videos. Six entropy measures were employed for extracting suitable features from the EEG signals. Next, we evaluated our proposed models using three popular classifiers: a support vector machine (SVM), multi-layer perceptron (MLP), and one-dimensional convolutional neural network (1D-CNN) for emotion classification; both subject-dependent and subject-independent strategies were used. Our experiment results showed that the highest average accuracies achieved in the subject-dependent and subject-independent cases were 85.81% and 78.52%, respectively; these accuracies were achieved using a combination of the sample entropy measure and 1D-CNN. Moreover, our study investigates the T8 position (above the right ear) in the temporal lobe as the most critical channel among the proposed measurement positions for emotion classification through electrode selection. Our results prove the feasibility and efficiency of our proposed EEG-based affective computing method for emotion recognition in real-world applications.

## 1. Introduction

Emotions play an essential role not only in human communication, but also in human perceptions; emotions also influence decision making [1]. Researchers have paid considerable attention to studying emotions in a vast number of interdisciplinary fields [2], such as affective neuroscience, computer science, psychology, and sociology of emotions. However, research on identifying emotional states automatically and accurately remains largely unexplored. Affective computing, also known as emotion AI [3], applies modern technology of human–computer interactions to human emotions [4]. Emotion AI has been proposed and developed in various applications, especially marketing, healthcare, driver assistance, and education [5]. In the field of marketing, emotion AI would include measuring customer reactions to products and services to optimize strategies, satisfy customers, and enhance productivity and profits [6]. An excellent example of the application of affective computing in healthcare is the early detection of a negative emotional state while reading social media news [7]. The extensive development of the Internet has led to the appearance of social media sites that provide users with convenient and quick access to information sources. However, young people are particularly vulnerable to inappropriate content that can lead to emotional disorders with prolonged exposure. Therefore, the development of emotion recognition systems is essential for preventing undesirable exposure to toxic online content. Some other applications of emotion recognition discussed in other studies are as follows. N. Murali Krishna et al. [8] described an approach based on the generalized mixture distribution model to identify the emotion recognitions by mentally impaired persons. Justas Salkevicius et al. [9] developed a virtual reality exposure therapy (VRET) system for handling multiple anxiety disorders using physiological signals like blood volume pressure (BVP), galvanic skin response (GSR), and skin temperature. However, the familiar point of these studies is that they all employ available datasets or apply expensive commercial devices with big sizes for data acquisition, so it is hard to be suitable for specific applications. Our study proposes a self-made device with compact size and lightweight that can be worn for a long time, verified in terms of data acquisition in our experiments.

Studies have shown that multiple factors can be used to recognize emotions, such as facial expressions, heart rate, body temperature, and electromyography [10,11,12,13,14]. Electroencephalography (EEG) signals have drawn considerable attention because of their high efficiency and objectivity [15]. In addition, EEG is a fast and non-invasive method [16] that provides reasonably precise brain responses to internal or external stimuli, especially for emotional stimuli [17]. Most previous investigations employed commercial equipment with a high cost and large size; in this research, we investigated and developed a low-cost, compact, wireless, and wearable device to collect EEG signals. However, electromagnetic interference is common in EEG data collection [18]. Therefore, signal preprocessing is vital to enhance system performance. Numerous studies have verified the success of the entropy measures because of the system’s sensitivity to variations in the physiological signal properties [19]. Furthermore, researchers have applied the entropy concept to quantify the amount of uncertainty or randomness in the pattern [20], which is exceptionally suitable for signals, such as EEG, which contain a large amount of information. In this study, six varieties of entropy measures were performed in both the time and frequency domains for feature extraction: permutation entropy (PEE), singular value decomposition entropy (SVE), approximate entropy (APE), sample entropy (SAE), spectral entropy (SPE), and continuous wavelet transform entropy (CWE).

Several previous studies have proved that not all electrodes employed for collecting EEG signals contained valuable information for emotion recognition [21]. Consequently, some electrode selection approaches were proposed to drop the EEG electrodes that contained unnecessary content [22]. This helped to decrease the computational complexity and boost the system speed. One popular selection technique is to combine the analysis of variance (ANOVA) [23] and Tukey’s honestly significant difference (HSD) tests [24]; we have followed this methodology in our study. We explored T8 as the preferred measurement position for EEG acquisition that holds valuable information for emotion recognition through electrode selection. In the last section of our system, we implemented the classification models that take on the role of automatically and precisely identifying the emotional states. In this study, we evaluated the performance of our proposed system using three popular classifiers: support vector machine (SVM), multi-layer perceptron (MLP), and one-dimensional convolutional neural network (1D-CNN). Moreover, we enhanced the processing speed by applying and weighing three dimensionality reduction techniques: principal component analysis (PCA), t-distributed stochastic neighbor embedding (t-SNE), and uniform manifold approximation and projection (UMAP). In addition to the benefits of price, portability, and ease of use, we also examined our wireless and wearable EEG device for feasibility and reliability through eye-closed and eye-opened testing. Moreover, with the obtained results from this study in emotion recognition, our system proved its ability to be employed for various purposes toward human life and the research community, such as entertainment, e-learning, or e-healthcare purposes.

In summary, the significant contributions of this study are as follows:

- We proposed a novel end-to-end affective computing based on machine learning for emotion recognition utilizing a wireless and wearable custom-designed EEG device with a low-cost and compact design.
- The combination of sample entropy feature extraction and 1D-CNN was introduced and achieved the best performance among the proposed entropy measures and machine learning models for two kinds of EEG emotion recognition experiments, including the subject-dependent and subject-independent cases.
- We also investigated that T8 in the temporal lobe was adopted most frequently through the electrode selection method, implying that this position would have further valuable information than other locations for emotion recognition.

The remainder of this paper is organized as follows: Section 2 describes the materials and methods. Next, the experimental results and the outcome of this study are presented in Section 3. Finally, Section 4 presents the conclusions of the paper.

## 2. Materials and Methods

### 2.1. Eight-Channel EEG Recording

**Figure 1:**
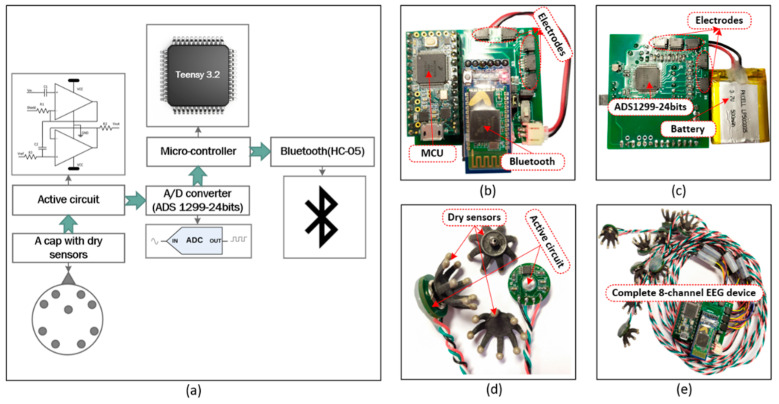
(**a**) Block diagram of the proposed EEG device; (**b**) top view of the EEG device; (**c**) bottom view of the EEG device; (**d**) dry sensors and active circuits; (**e**) complete 8-channel EEG device.

**Figure 2:**
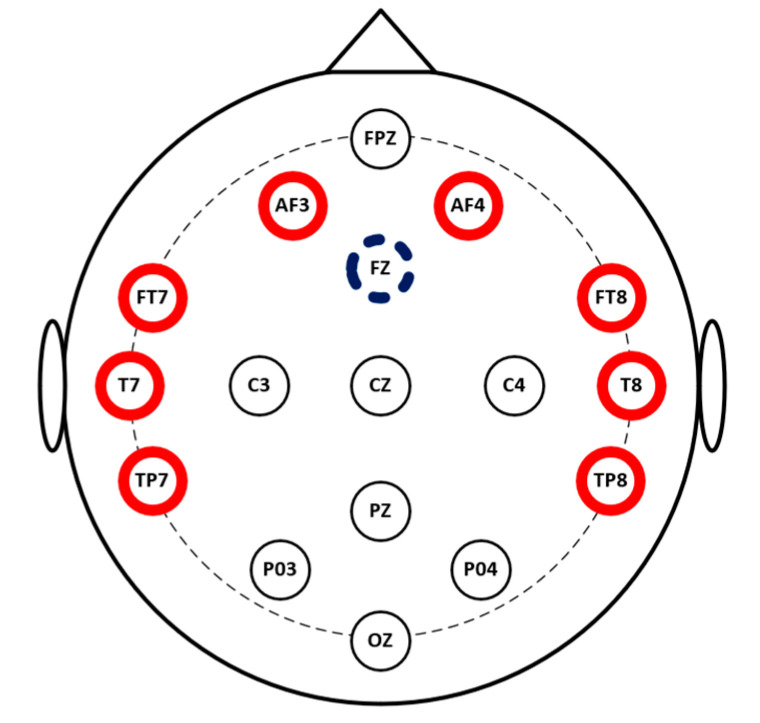
Two frontal electrodes (AF3 and AF4) and six temporal electrodes (FT7, FT8, T7, T8, TP7, and TP8) selected in the study.

In this study, we developed a complete EEG-based affective computing method for emotion recognition. The system consists of two main parts: an EEG headset and the software. The main function of the headset is to collect EEG signals at the sampling rate of 256 Hz from the 8-channel electrode cap. Then, the headset transfers the signals to the software set up on a computer so that all the data are managed and processed using the Bluetooth wireless protocol. The proposed EEG device is shown in Figure 1. We used “dry” active sensors with pre-amplification circuits to solve the high electrode–skin interfacial impedances when collecting EEG signals from the brain through the hair. In this study, we used sensors in the range of 100–2000 kOhm on the unprepared leathers produced by Cognionics, Inc., San Diego, USA [25]. ADS 1299 is an 8-channel, 24-bit analog-to-digital converter that specializes in EEG applications [26] used to digitalize the obtained signals. Before the digitized data were sent to a computer via the wireless protocol using the HC-05 Bluetooth module, the data were conveyed to the Teensy 3.2 board used as a microcontroller. The EEG data were gathered and displayed continuously by self-programmed and pre-installed software on our computer. Previous studies have explained how the frontal and temporal lobes are critical brain areas for research on emotions [27,28,29]. Therefore, we collected EEG signals from the two frontal electrodes (AF3 and AF4) and the six temporal electrodes (FT7, FT8, T7, T8, TP7, and TP8) in this study that were placed on the scalp according to the international 10–20 system [30] (see Figure 2).

**Figure 3:**
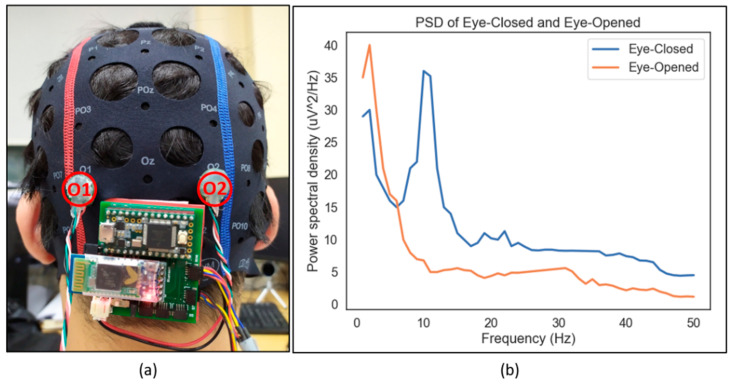
(**a**) Experimental setup for EEG device testing; (**b**) power spectral densities (PSDs) during eyes-closed and eyes-opened resting state from O1-O2 locations.

There are multiple measures to evaluate the feasibility of a device for EEG acquisition, such as using the attention/meditation level or eye blinking or eye-closed and eye-opened testing [31]. This study assessed our device through eye-closed and eye-opened resting-state signals (the outcomes are shown in Figure 3). Neural alpha wave is identified in delivering a dominant peak of approximately 10 Hz in the occipital cortex during closing eyes. O1–O2 positions are used to collect EEG signals from the occipital cortex. Welch’s periodogram is adopted to compute PSD (Power Spectral Density) with a 1-s (256 data points) window and a 50% overlap.

### 2.2. Stimulus and Protocol

It is important to design reliable stimuli for emotion elicitation. Different stimulants have been employed in emotion investigations in recent years, such as images, music, movies, and games. Databases, such as International Affective Picture System [32], Database for Emotion Analysis Using Physiological Signals [33], Geneva Affective Picture Database [34], Nencki Affective Picture System [35], and EmoMadrid [36], have been used. Studies have underlined the importance of the practicability and effectiveness of images in discovering emotional states [37,38,39]. In our experiments, we selected pictures from the EmoMadrid dataset to produce emotions in the participants. This dataset contains over 700 photos across a wide variety of genres; the dataset contents have normative ratings for three categories of emotions: negative, neutral, and positive [36]. We carefully chose images from this dataset to create 30 videos, and the same number of images were shown to each participant for each of the three emotional states.

**Figure 4:**
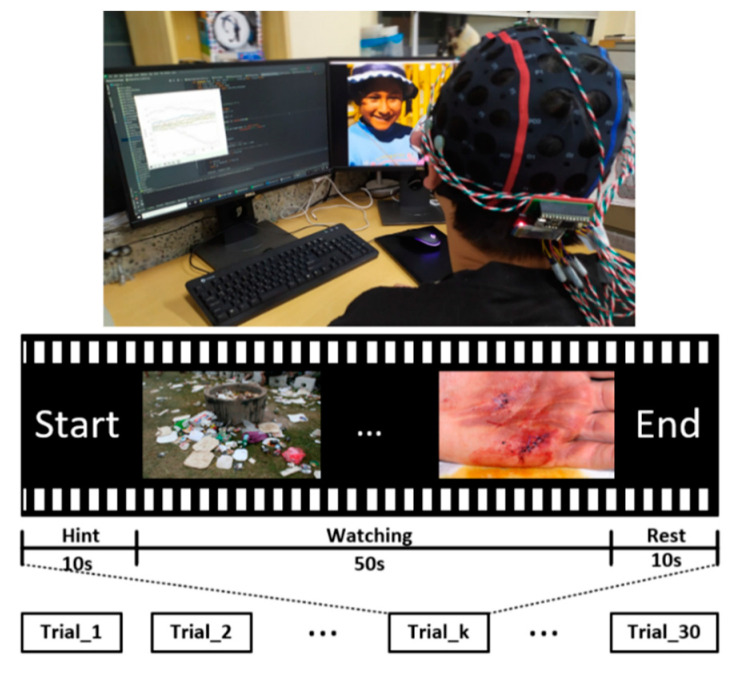
Protocol of the EEG experiment.

A total of eight persons (all males) in the age group of 25 to 40 years participated in this study. They were all students from Pukyong National University, Busan, Korea. Some previous studies revealed little or almost no significant difference in the gender-based emotion classification on EEG signals [7,40,41]. Hence, in this study, we will not concentrate on judging the impact of gender on EEG signals in emotion recognition. All the participants were both physically and mentally healthy without any eyesight problems. They were instructed not to use any stimulants, such as alcohol or drugs, a day before the experiment to avoid any adverse effects on the test accuracy. Note that the experiments on human subjects were performed by the approval of the Institutional Review Board at Pukyong National University for biomedical research. Each participant read a clear description of the research paper and agreed to sign a consent form. They were comfortably seated in an upright position in front of the computer screen, and they wore the EEG headsets. In each experiment, a participant watched 30 short videos. In each video, 10 s were given for the hint, 50 s for viewing pictures, and 10 s for rest. Figure 4 shows the detailed protocol.

### 2.3. Feature Extraction

The obtained EEG signals were cleaned with a band-pass filter at 1–50 Hz to reject all unwanted noise. For a trial of the signal collection process, the EEG signals were segmented with a sliding window of 0.5 s (128 data points) having an overlap rate of 50%; this segmentation ensured that there was no lack of valuable information because of the temporal correlation of the data points. Next, we applied the six variants of entropy measures to extract useful features from the preprocessed EEG data for classifying the emotional states. Entropy is a measure of uncertainty. The level of disturbance in biomedical signals, including EEG, can be estimated by employing the system’s entropy. A higher entropy implies more significant uncertainty and a more chaotic approach. Entropy is given as follows:

(1) $$\mathrm{Entropy}=x\left(n\right)\int_{\min\left(x\right)}^{\max\left(x\right)}P_{x}\log\left(\frac{1}{P_{x}}\right)d_{x},$$

where P_x_ is the probability density function of the signal x(n).

#### 2.3.1. Permutation Entropy (PEE)

PEE [42] is a robust nonlinear statistical tool for time-series data, which determines the order relationships between its values to measure quantitative complexity of a dynamic system. Then, this tool extracts the probability distribution of a set of ordinal patterns. Some notable features of the method are non-parametric, robust, flexible, and computationally efficient.

#### 2.3.2. Singular Value Decomposition Entropy (SVE)

SVE [43] symbolizes the numerous eigenvectors required for a sufficient description of the time-series data. SVE is a powerful tool to measure the signal uncertainty, and it has a vast and vital application in signal processing.

#### 2.3.3. Approximate Entropy (APE)

APE [44] is a technique used to quantify the volume of consistency and the unpredictability of changes across the time-series signals. It was first introduced as a variant of the Kolmogorov–Sinai entropy by Steve M. Pincus in 1991 to investigate medical signals, such as heart rate and EEG. Then, it was widely implemented in multiple diverse fields, including economics, physiology, and climate sciences.

#### 2.3.4. Sample Entropy (SAE)

SAE [45] is an improvement over APE; it eliminates the existing limitations of APE. Richman and Moorman proposed assessing the complexity of the physiological time-series signals with data length independence and achieved relatively trouble-free implementation.

#### 2.3.5. Spectral Entropy (SPE)

SPE [46] is a measure of the signal irregularities in Shannon entropy. Most physiological signals, including EEG signals, are nonlinear; therefore, SPE as a nonlinear method is ideal for analyzing neural signals. It employs spectral analysis methods, such as the Fourier transform and Welch periodogram, to transform signals from the time domain to the frequency domain. Then, the SPE method implements the Shannon entropy concept to distribute the spectral power.

#### 2.3.6. Continuous Wavelet Transform Entropy (CWE)

Wavelet transform (WT) [47] is considered the most accurate approach for time-frequency analysis to overcome the constraints of short-time Fourier transform in window size selection. The WT overcomes the drawbacks by decomposing the signal in both the time and frequency domains for multiple resolutions. It applies a modulated window moved along with the signal at various scales. In this study, we adopted a continuous WT using Morlet for EEG signal analysis and obtained the probability distributions of each of the individual wavelet energies and the total wavelet energy. Then, these distributions are used to characterize the Shannon entropy.

### 2.4. Electrode Selection

EEG signals were collected from multiple electrodes attached to the various measurement positions on the scalp. However, not all the obtained data from the measurement positions provided critical and necessary information; in fact, the data collected depended on the purpose of the particular study. Various studies focus on the issue and determine the optimal electrode locations for emotion recognition. Sofien Gannouni et al. [48] described a zero-time windowing-based proper electrode selection method to identify emotional states. We used ANOVA to select electrode locations with crucial content for emotion classification. ANOVA was developed by Sir Ronald A. Fisher (1925) [23]; it is a statistical technique to determine whether there are any statistically significant differences between the mean values of three or more independent groups. However, one drawback of using ANOVA was that it could not detect the specific difference between each pair of groups [49]. To obtain details about the differences between any two groups, we need to append Tukey’s HSD test [24], known as the mathematical test, for obtaining mean values significantly distinct from each other. This study employs ANOVA and Tukey’s HSD test as an electrode selection method for each feature type and each measurement location to determine whether there is a significant difference in emotion classification between each pair of emotional states (Positive–Negative, Negative–Neutral, and Neutral–Positive). Then, a decision is made to select or reject the measurement position. This method helps lessens the computational complexity of the models, which boosts the system’s speed.

### 2.5. Dimensionality Reduction

Data with high dimensionality introduces computational complexity into the classification process and yields unexpectedly low results. For real-world applications, dimensionality reduction aims to convert data from a high-dimensional space into a low-dimensional space to increase the classifier’s speed and stability. An advantage of dimensionality reduction is to make data visualization more apparent using a two- or three-dimensional space; this visualization becomes a complex challenge when a large number of dimensions are involved. In this study, we compare three popular approaches: PCA, t-SNE, and UMAP.

PCA [50] is considered a statistical technique that enables the extraction of information content from an extensive original dataset into an uncorrelated variable called the principal component. PCA applies a linear transformation to arrange the components with the highest possible variance generated from the input data. PCA reduces the dataset’s dimension and identifies the artifactual components from the EEG signals to eliminate the unwanted signals.

Additionally, t-SNE [51] is a nonlinear technique for dimensionality reduction; it is a helpful tool for the visualization of high-dimensional datasets. It was developed in 2008 by Laurens van der Maatens and Geoffrey Hinton; t-SNE calculates the similarities between the pairs of instances in high-dimensional space and low-dimensional space. PCA is employed for maximizing the variance with large pairwise distances, whereas the t-SNE would only consider small pairwise distances or local similarities.

UMAP [52] is an algorithm based on manifold learning techniques; this algorithm depends on the ideas obtained from topological data analysis for data reduction. In application and research, UMAP is superior to t-SNE because of its faster processing speed. Furthermore, UMAP tends to work on data-dimensionality reduction, which is suitable for machine learning preprocessing, whereas t-SNE is mainly suitable for data visualization.

### 2.6. Classification

#### 2.6.1. Support Vector Machine (SVM)

SVM is a simple classifier [53] that finds an optimal hyperplane between the data of two classes so that the distance between the closest data points to that hyperplane is the greatest. The nearest points are called the support vectors, whereas the distance that needs to be maximized is called the margin. The SVM is a binary classifier that can be extended into a multi-class classifier by using the one-to-rest approach. This approach determines a hyperplane that separates a class from all other classes at once. This study employs the radial basis function, which is most commonly used as a kernel in SVM.

#### 2.6.2. Multi-Layer Perceptron (MLP)

**Figure 5:**
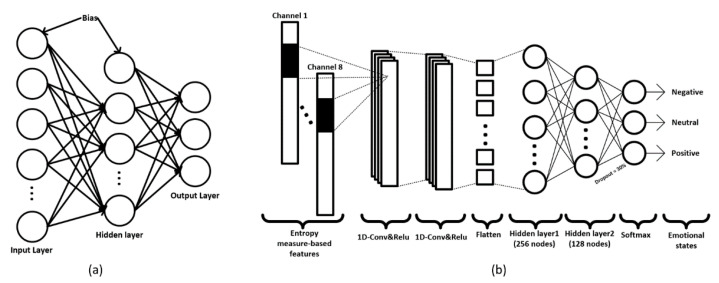
(**a**) The proposed MLP model; (**b**) the proposed 1D-CNN model.

MLP is a feed-forward artificial neural network (ANN) pattern [54] known as a “vanilla” neural network because it possesses only one hidden layer. We propose an MLP model with three layers: one input layer, one hidden layer, and one output layer (see Figure 5a). The size of the input layer is that of each extracted feature. The hidden layer consists of 128 neurons followed by the rectified linear activation unit (ReLU), which executes a ReLU nonlinear function. Finally, the SoftMax function, which is a normalized exponential function, is used to normalize a network’s output to a probability distribution over the predicted output classes.

#### 2.6.3. 1-D Convolutional Neural Neworks (1D-CNN)

Convolutional neural network (CNN) [55] is a great ANN technique applied in various fields ranging from image classification to bio-signal processing. CNN models are widely used when working with images in which the input of the model is two- or three-dimensional data that represent the pixels and channels of the pictures. 1D-CNN, as a variant of CNN, is also often applied to one-dimensional data sequences suitable for biomedical signals, such as EEG, electrocardiogram, and heart rate. We propose a 1-D CNN model with a 1D-Convolution (1D-CONV) layer with 256 neurons and two hidden layers with 128 neurons each (see Figure 5b). The 1D-CONV filter is considered the most critical part of 1D-CNN because it uses kernels to convolve the input data. In MLP and 1D-CNN, we initiated the supervised learning rate as 0.001, the optimizer as the Adam, and the loss function as the categorical cross-entropy.

## 3. Results

**Figure 6:**
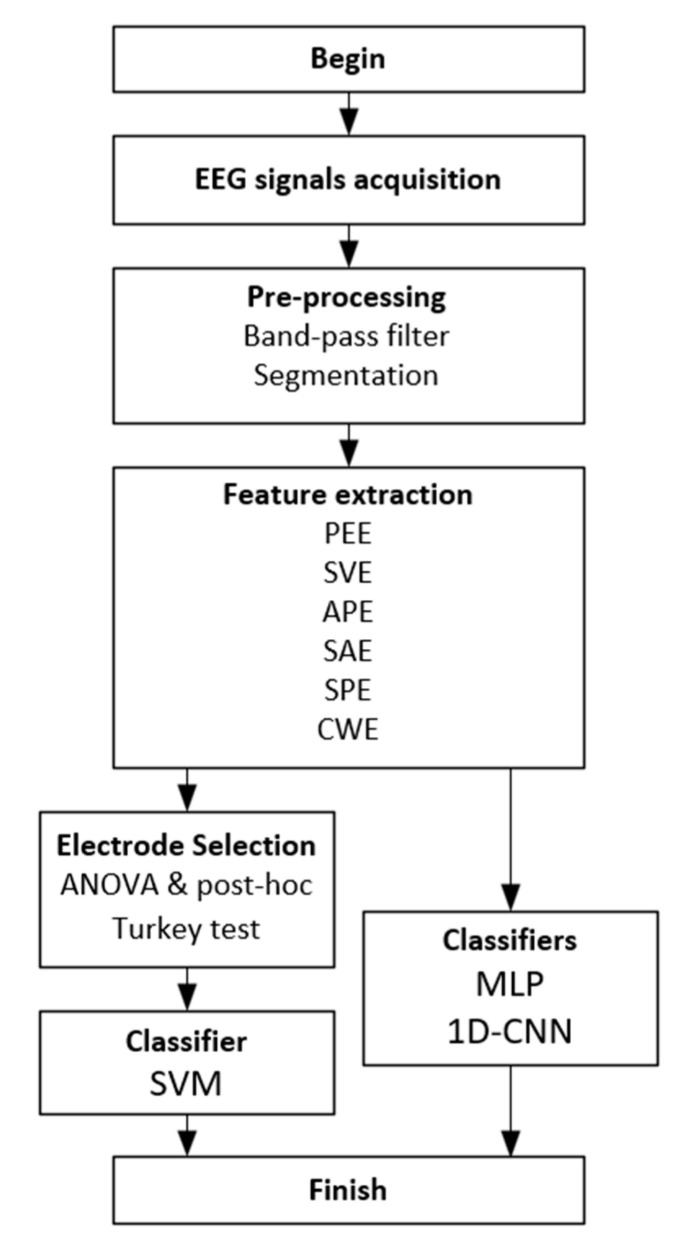
Workflow diagram of the proposed system.

Figure 6 shows the workflow diagram of our proposed system. The methods and algorithms used in this study are described concisely in each block.

### 3.1. EEG Features

First, we applied the band-pass filter (1–50 Hz) for the raw EEG signals. Then, before using the six entropy measures for feature extraction, we split the EEG signals into segments of 0.5 s with an overlapping rate of 50%. The entropy measures used in this study include PEE, SVE, APE, SAE, SPE, and CWE. The assessed values after feature extraction are normalized from 0 to 1 to promote computation in the following steps.

**Figure 7:**
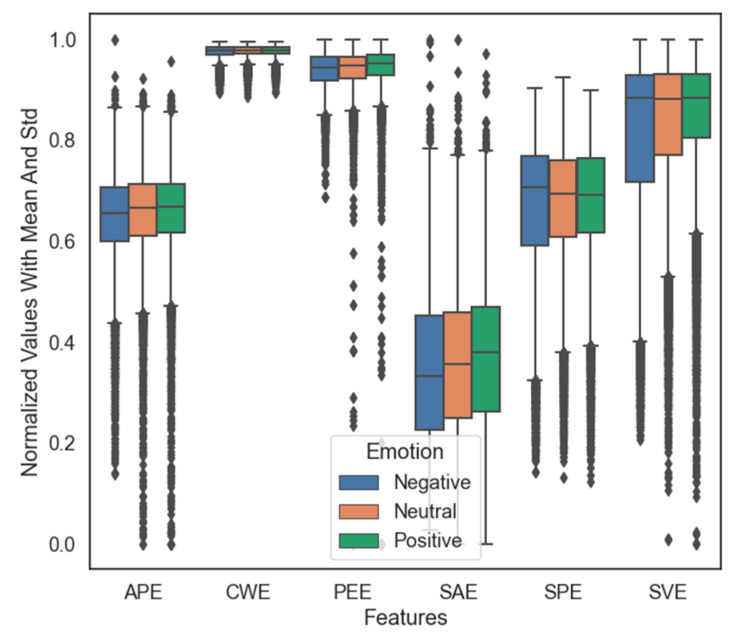
Normalized values with the mean and standard deviation for the six features of the three emotions.

Figure 7 presents the mean and the standard deviation values of the different entropy measures for three emotional states. From Figure 7, we can see that the effect of each feature type is distinct in both magnitude and the distribution of values. PEE and CWE have the highest mean values, and their standard values are the least. In contrast, SAE has a minor mean, and its standard deviation is the highest. Nevertheless, by depending only on their mean and standard deviation values, it is difficult to determine which of these six features are suitable for emotion recognition.

In this study, each participant watched a total of 30 videos for eliciting their emotions, 10 videos for each type of emotional state corresponding to 3 labels (Negative, Neutral, and Positive). Each video lasts 50 s. Data segmentation is done with a 0.5 s window and 50% overlap. Therefore, in the case of 30 videos per participant, we have 30 (videos) ∗ 8 (channels) ∗ [50 s (each video) ∗ 2 (0.5 s window) ∗ 2 (50% overlap) − 1] = 47,760 segments utilized for feature extraction. After feature extraction, 8 (channels) ∗ 5970 (features) are used as the dataset for building models. We apply 5-fold cross-validation for training, validation, and evaluation of the models in the subject-dependent case. Additionally, in the subject-independent case, we employ leave-one (subject)-out cross-validation, which handles a subject’s sample data as a test set for evaluating the model after it has been trained and validated on the data sample of the remaining subjects.

### 3.2. EEG Electrode Selection and Rejection

**Figure 8:**
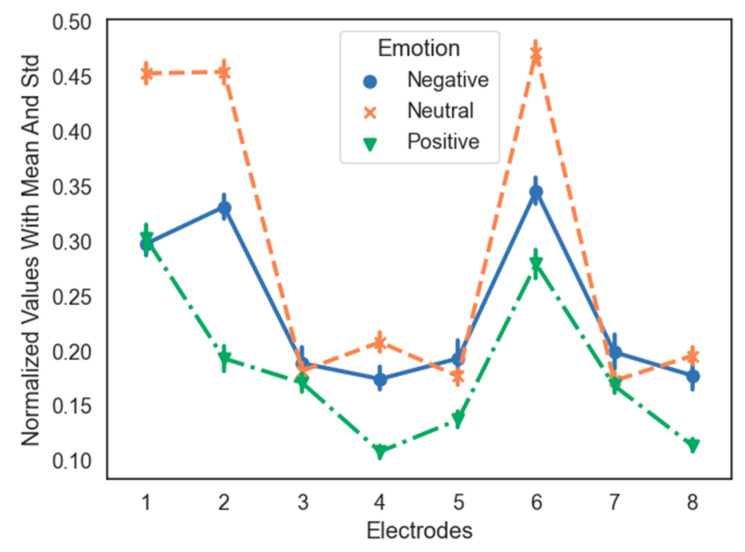
Normalized values with the mean and standard deviation of three emotional states across each electrode using SAE on Subject 2.

Not all the EEG signals obtained from the electrode positions on the head provide the information necessary for emotion classification. Electrode selection not only eliminates irrelevant noise, but also speeds up the computation of classifiers. Some previous studies have focused on identifying the optimal electrode placement in emotion recognition. In this study, we used ANOVA and Tukey’s HSD test for electrode selection. Figure 8 describes the distribution of the data points of three emotional states over each electrode. It is not complicated to determine the electrode that provides helpful information about the significant differences between the three emotional states. Table 1 lists the *p*-values of the various pairs of emotional states on all the eight channels and shows which position needs to be chosen or removed. For each electrode to be selected, no pair of emotional states should have a *p*-value greater than 0.05. For example, for electrode 1 (placed on the AF3 location), the *p*-value of the negative–neutral and neutral–positive groups is equal to 0.001 (*p* < 0.05). However, the *p*-value of the negative–positive couple was 0.8130 (*p* > 0.05), which implied that there was no significant difference between these two groups; therefore, we removed the AF3 location.

**Figure 9:**
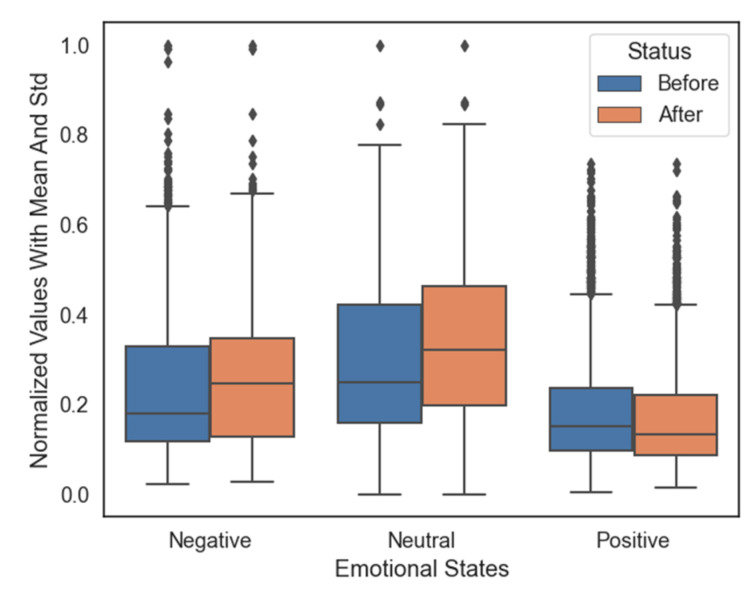
Normalized values with the mean and standard deviation values across the three emotional states before and after electrode selection using SAE on Subject 2.

**Figure 10:**
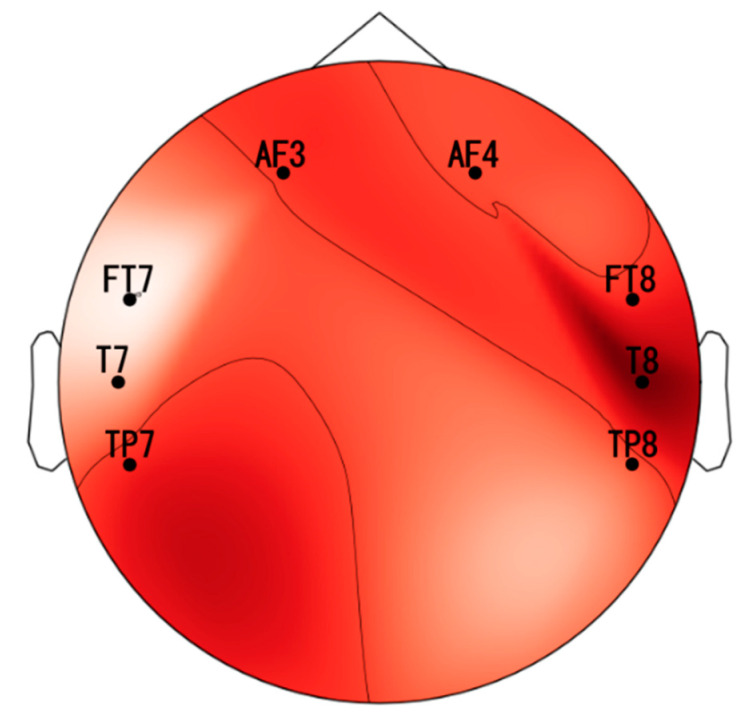
Topographical scalp map of the selected EEG electrodes.

Figure 9 shows notable variations in the mean and standard deviation values of the data before and after removing the unnecessary electrodes. The average values of the negative and neutral groups increase simultaneously; however, the average value in the positive data group shows a decrease. We performed electrode selection on all the six features across the eight subjects (see Table 2). The outcomes show that the selected electrodes depended on the types of features, and the outcomes varied from subject to subject. Figure 10 shows the rate of selection of electrodes using a topographical plot. The dark position shows the areas where more electrodes are preferred. Clearly, the T8 in the temporal lobe has been chosen most frequently, which implies that this position would have more helpful information than other positions for classifying the emotional states.

### 3.3. Classification Results

**Figure 11:**
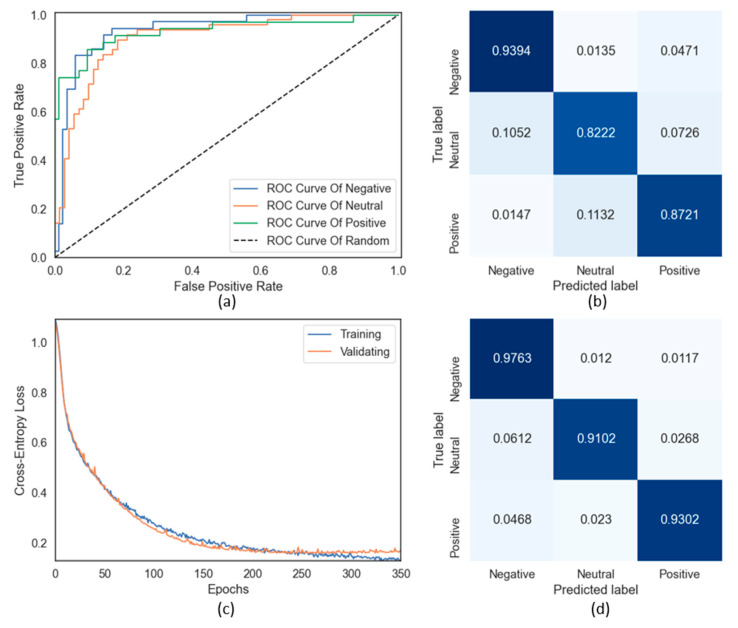
The results for SAE feature on Subject 2: (**a**) ROC curves for the SVM classifier; (**b**) normalized confusion matrix of the SVM classifier; (**c**) loss curve of the proposed 1D-CNN; (**d**) normalized confusion matrix of the proposed 1D-CNN.

The features extracted after using electrode selection would be accepted as the input to the SVM classifier. Figure 11b shows that the accuracy result obtained by using SAE from Subject 2 is 87.79%; it is described in detail with the confusion matrix. Accuracy, sensitivity, and specificity are calculated as follows based on the confusion matrix:

(2) $$\mathrm{Accuracy}=\frac{\mathrm{TP}+\mathrm{TN}}{\mathrm{TP}+\mathrm{TN}+\mathrm{FP}+\mathrm{FN}},$$

(3) $$\mathrm{Sensitivity}=\frac{\mathrm{TP}}{\mathrm{TP}+\mathrm{FN}},$$

(4) $$\mathrm{Specificity}=\frac{\mathrm{TN}}{\mathrm{TN}+\mathrm{TP}},$$

where TP, TN, FP, and FN represent the true positive, true negative, false positive, and false negative, respectively.

To visualize the performance of the multi-class classification problem, we also used the area under the curve (AUC)–receiver operating characteristics curve [56], as a graphical plot using the true positive rate against the false positive rate at various threshold settings. When the AUC increases, the model will have greater capacity to distinguish between classes. In this case, the AUC with SVM results for the three categories negative, neutral, and positive are 0.93, 0.90, and 0.92, respectively, as shown in Figure 11a. Table 3 lists the classification results using SVM for all the six features; the highest average accuracy using SAE across eight subjects is 79.36%.

This study also employs two additional state-of-the-art classifiers, MLP and 1D-CNN, for EEG-based emotion recognition. To evaluate the performances of the MLP and 1D-CNN models, we used the extracted features without using electrode selection as the input data to keep all the information from all electrodes. As discussed earlier, electrode selection is applied to hold relevant information and reject unnecessary information for emotion recognition. Thus, it makes model computation less complicated and faster, suitable for traditional machine learning models like SVM. However, from Table 1, we take the rejected electrodes into deep consideration. Take electrode 1 as an example; it is dropped because there is no notable difference between the negative–positive pair (*p* = 0.8130 > 0.05); however, the two remaining pairs still meet the condition *p* < 0.05. Hence, if removing these electrodes, there is a high probability that critical information for classification will disappear, causing the system’s accuracy to be reduced. Moreover, neural networks are well-known for the advantages of fast computation and the ability to work competently in extracting valuable features from the input data. Therefore, in the case of MLP and 1D-CNN, we take all the features obtained as input to these models to ensure that no vital information is lost, which increases the system’s accuracy. Using 5-fold cross-validation, the proposed models were trained and evaluated in the study. We split the dataset into five subsets of which one subset was used as the test set; the other four subsets were adopted for training the model. This process was iterated over each subset. The final result was the average of the performance accuracy on each subset. Table 3 shows that the highest average results using the three proposed models SVM, MLP, and 1-CNN over eight subjects are 79.36%, 81.52%, and 85.81%, respectively; all the models used SAE as the feature input. Figure 11c shows the loss curve and confusion matrix of 1D-CNN’s performance using SAE on Subject 2; this model achieves the highest accuracy of 93.89%.

### 3.4. Subject-Dependent and Subject-Independent

**Figure 12:**
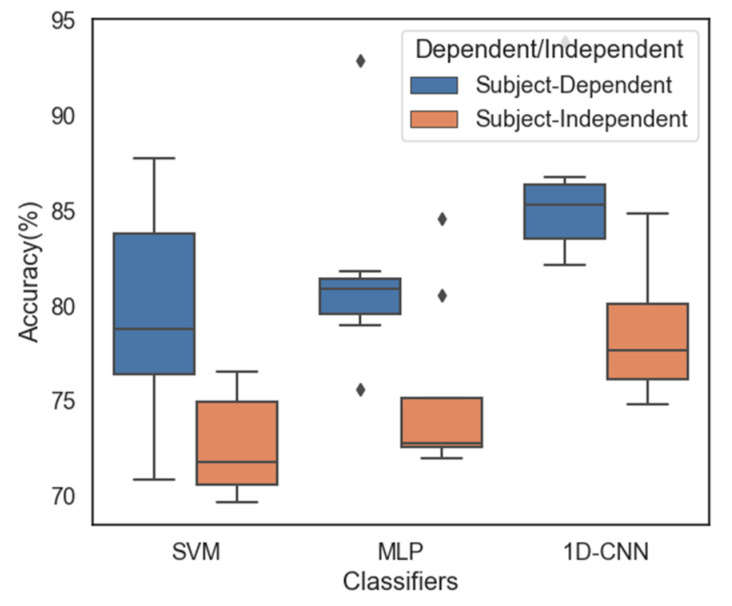
Comparison of the classification accuracies of the three classifiers for three emotions with both subject-dependent and subject-independent cases for eight subjects using SAE.

To make our system more practical for real-world applications, we trained the proposed models across all subjects with the data used for the subject-independent strategy. For the subject-dependent case, we developed a new model consisting of the training and testing data from each subject’s dataset; high accuracy was achieved by this method, but it could not be generalized. For the subject-independent strategy, we used a subject’s data sample for testing the proposed models, and the datasets of the remaining subjects were used for training. Figure 12 shows a comparison of the results obtained for subject-dependent and subject-independent cases using SVM, MLP, and 1D-CNN with SAE. As mentioned, the average accuracies achieved in the subject-independent case using SVM, MLP, and 1D-CNN with SAE were 72.65%, 75.14%, and 78.52%, respectively; these values are lower than the average accuracies obtained for the subject-dependent case (79.36%, 81.52%, and 85.81%, respectively). Even with this comparatively high level of accuracy, the subject-independent models demonstrated that our proposed system for EEG-based emotion recognition is practically feasible.

### 3.5. Dimensionality Reduction and Data Visualization

**Figure 13:**
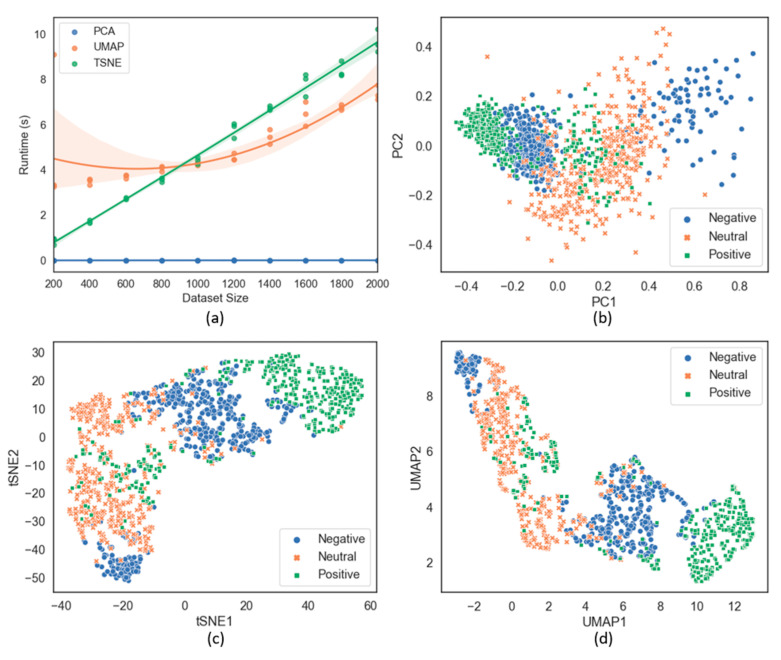
(**a**) Runtime of three dimensionality reduction techniques (PCA, t-SNE, and UMAP). (**b**) Data visualization after using PCA with n_components = 2, svd_solver = auto. (**c**) Data visualization after using t-SNE with n_components = 2, metric = ‘Euclidean’. (**d**) Data visualization after using UMAP with n_components = 2, metric = ‘Euclidean’. Data were obtained by using SAE on Subject 2.

**Figure 14:**
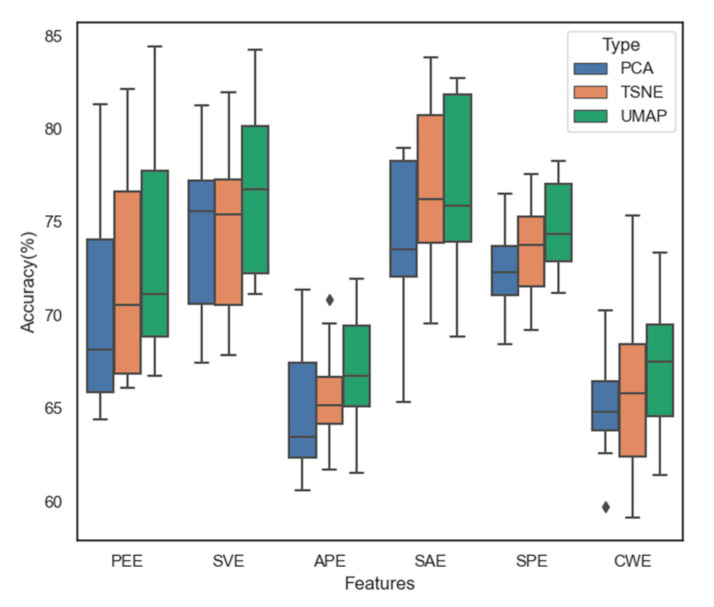
Comparison of classification accuracy of PCA, t-SNE, and UMAP with SVM using all features across eight subjects.

Speed is one of the most critical factors for assessing whether or not a system is feasible in real-world applications. Dimensionality reduction helps reduce the computational complexity for classifiers and thereby improves their stability. In addition, dimensionality reduction is a helpful tool for data visualization, which becomes challenging with multi-dimensional data. In this study, a comparative analysis is made of the three approaches PCA, t-SNE, and UMAP to reduce the number of EEG data dimensions. We evaluated the processing speed of each method. Figure 13a shows the runtime values of the three approaches when they are applied to the resampled dataset using SAE on Subject 2. Dimensionality reduction was performed three times for each subject using multiple dataset sizes to ensure that the evaluation was objective. PCA is always the fastest option for all different dataset sizes. When working on small datasets, t-SNE is more robust than UMAP. However, as the dataset size gradually increases, UMAP outperforms t-SNE. Figure 13b–d show the results obtained by applying the three methods. Based on the data visualization, it is clear that data distribution with UMAP is the most useful as compared with the other two classification methods. However, to precisely evaluate their performances in classifying EEG-based emotion states, we implemented these approaches for the datasets of the six types of features on eight subjects with SVM as a simple classifier. Figure 14 shows the obtained results. Although the PCA speed is the highest, it has the least accuracy for classification. UMAP has demonstrated its strength in its high speed and accuracy on all six types of features across eight subjects; it has the highest average accuracy of 76.78% with SAE. Therefore, UMAP will be suitable for real-world emotion recognition applications.

## 4. Conclusions

This study proposed an EEG-based affective computing method for emotion recognition with three categories of emotions (negative, neutral, and positive). We collected 8-channel EEG signals using our wireless and wearable custom-designed device. A total of 30 trials were conducted on eight participants while they watched emotive videos. In this study, six entropy measures were employed for feature extraction, and three standard classifiers (SVM, MLP, and 1D-CNN) were implemented for emotion classification. After feature extraction, we classified the emotion states by electrode selection to retain all the important information and remove all the unnecessary information. The results proved that T8 in the temporal lobe contained much valuable content for emotion recognition.

We completed the classification process with subject-dependent and subject-independent strategies using six features and three classifiers across eight subjects. The accuracy values of our proposed system for detecting emotion states as short as 0.5 s with subject-dependent and subject-independent strategies were 85.81% and 78.52%, respectively; we used a combination of SAE and 1D-CNN.

To meet the requirements of high-speed processing and stable accuracy during practical applications of a system, we compared three approaches (PCA, t-SNE, and UMAP) for dimensionality reduction and applied SVM as a classifier. The achieved results demonstrated our proposed system’s feasibility in real-life applications; the highest accuracy of 76.78% was obtained with UMAP.

Besides, our proposed system has some limitations that need to be considered in the following steps. First, although the number of experiments (30 trials) on each subject is moderately enough to create a complete dataset, the number of participants is still limited. Additionally, our research still has not focused on the impact of gender and age on the changes of EEG signals in emotion recognition. Hence, we will conduct experiments on more subjects and assess other aspects such as age and gender. Another issue in this work is that in the signal preprocessing, we currently apply a simplistic bandpass filter to eliminate unwanted signals out of the frequency range of 1 to 50 Hz. However, filtering like this has constraints in not handling different types of artifacts associated with the participant well, such as electromyography (EMG) caused muscle movements. Therefore, in the following study, we will implement more advanced and efficient techniques in signal processing such as blind source separation (BSS) or independent component analysis (ICA) to reduce eye movements, blinks, muscle, heart, and line noise. Furthermore, our future work will concentrate on building and deploying robust machine learning models on our embedded device and then connect to IoT-connected healthcare platforms; this makes our system straightforward and convenient to achieve in real-world applications.

## Figures and Tables

**Table 1:** EEG electrode selection for SAE over Subject 2.

Electrode	*p*-Value (ANOVA and Tukey’s HSD Test)			Decision (*p* ≤ 0.05)
	neg-neu	neg-pos	neu-pos	
1	0.001	0.8130	0.001	reject
2	0.001	0.001	0.001	adopt
3	0.5900	0.0365	0.2916	reject
4	0.001	0.001	0.001	adopt
5	0.1053	0.001	0.001	reject
6	0.001	0.001	0.001	adopt
7	0.0029	0.001	0.8367	reject
8	0.0225	0.001	0.001	adopt

Note that: 1-AF3, 2-AF4, 3-FT7, 4-FT8, 5-T7, 6-T8, 7-TP7, 8-TP8.

**Table 2:** EEG electrode selection for six entropy measures across eight subjects.

Subject	Selected Electrodes					
	PEE	SVE	APE	SAE	SPE	CWE
1	1, 3, 4, 5, 6, 7, 8	6, 7	2, 4, 5, 6, 8	4, 5, 6, 7, 8	1, 2, 3, 6, 7	1, 2, 6, 7
2	2, 5	1, 3, 4, 5, 8	2, 4, 5, 8	2, 4, 6, 8	2, 4, 5, 6, 8	1, 2, 4, 5, 6
3	4, 8	All electrodes	3, 4, 6	1, 2, 3, 4, 6, 7, 8	1, 2, 3, 4, 7, 8	2, 4, 6, 7
4	6, 7	1, 6	4, 6	1, 7	1, 3, 6	1, 2, 6
5	1, 3, 4, 5, 6, 7, 8	2, 4, 6	2, 4, 6, 7	2, 3, 4, 6, 7	1, 2, 5	6, 8
6	5, 6, 7	1, 2, 4, 5, 6, 7, 8	4, 6	1, 2, 4, 5, 6, 7, 8	1, 2, 5, 6, 7, 8	1, 3, 6, 8
7	4, 8	1, 2, 4, 5, 6, 7, 8	7, 8	3, 4, 7, 8	7, 8	1, 4, 6
8	2, 5, 6	1, 3, 7	1, 7	1, 2, 7, 8	1, 7	1, 2, 5

Note that: 1-AF3, 2-AF4, 3-FT7, 4-FT8, 5-T7, 6-T8, 7-TP7, 8-TP8.

**Table 3:** Comparison of classification accuracy (%) between SVM, MLP, 1D-CNN with six features across eight subjects.

Subject	EEG Features								
	PEE			SVE			APE		
	SVM (%)	MLP (%)	1D-CNN (%)	SVM (%)	MLP (%)	1D-CNN (%)	SVM (%)	MLP (%)	1D-CNN (%)
1	83.33	70.22	79.64	73.14	74.14	81.97	69.17	78.33	75.88
2	85.93	76.54	83.46	81.87	70.28	77.78	74.92	72.59	75.39
3	69.45	67.92	69.44	87.52	89.72	90.35	65.89	65.83	66.58
4	68.57	70.04	70.65	72.99	71.03	82.22	67.67	68.22	68.56
5	75.92	75.65	80.98	81.58	78.17	84.05	74.27	75.56	76.25
6	70.54	71.39	71.27	81.25	79.72	84.62	66.95	67.84	68.01
7	71.75	72.45	71.55	79.49	79.04	83.70	63.47	65.48	69.16
8	68.98	71.13	70.42	72.67	79.80	84.44	68.50	69.57	68.09
**Mean**	74.31	71.92	74.68	78.81	77.74	83.64	68.85	70.43	70.99
**Std**	6.73	2.71	5.30	5.04	5.78	3.28	3.69	4.35	3.82
**Subject**	**EEG Features**								
	**SAE**			**SPE**			**CWE**		
	**SVM** **(%)**	**MLP** **(%)**	**1D-CNN** **(%)**	**SVM** **(%)**	**MLP** **(%)**	**1D-CNN** **(%)**	**SVM** **(%)**	**MLP** **(%)**	**1D-CNN** **(%)**
1	70.89	80.95	86.75	78.38	75.45	86.12	68.72	71.63	72.18
2	87.79	92.90	93.89	87.50	84.72	84.90	73.29	72.61	72.37
3	83.70	81.28	86.26	79.97	84.98	80.56	66.92	65.95	66.88
4	73.61	81.83	83.06	79.82	82.57	81.50	64.74	65.52	66.76
5	84.06	80.81	84.75	73.10	77.17	76.26	75.23	75.63	76.55
6	79.33	79.76	85.94	72.59	74.44	77.59	68.56	67.93	68.25
7	78.19	75.65	82.17	74.57	77.22	76.99	65.62	66.84	67.87
8	77.32	78.97	83.68	75.93	80.05	80.78	69.54	70.97	72.91
**Mean**	79.36	81.52	85.81	77.73	79.58	80.59	69.08	69.64	70.47
**Std**	5.27	4.67	3.41	4.56	3.87	3.37	3.39	3.38	3.31

Note: SVM was used for features with electrode selection whereas MLP and 1D-CNN were used for features without electrode selection.

## Data Availability

The data presented in this study are available on request from the corresponding author. The data are not publicly available due to their containing information that could compromise the privacy of research participants.
